# Nano-Modeling and Computation in Bio and Brain Dynamics

**DOI:** 10.3390/bioengineering3020011

**Published:** 2016-04-05

**Authors:** Paolo Di Sia, Ignazio Licata

**Affiliations:** 1 Department of Philosophy, Education and Psychology, University of Verona, Lungadige Porta Vittoria 17, Verona 37129, Italy; 2 ISEM, Institute for Scientific Methodology, Palermo 90146, Italy; Ignazio.licata@ejtp.info; 3 School of Advanced International Studies on Applied Theoretical and Non Linear Methodologies in Physics, Bari 70121, Italy

**Keywords:** neuro-nanoscience, cognitive science, bioengineering, brain, carrier transport, theoretical modeling, neural geometry, memristor, electrical circuits

## Abstract

The study of brain dynamics currently utilizes the new features of nanobiotechnology and bioengineering. New geometric and analytical approaches appear very promising in all scientific areas, particularly in the study of brain processes. Efforts to engage in deep comprehension lead to a change in the inner brain parameters, in order to mimic the external transformation by the proper use of sensors and effectors. This paper highlights some crossing research areas of natural computing, nanotechnology, and brain modeling and considers two interesting theoretical approaches related to brain dynamics: (a) the memory in neural network, not as a passive element for storing information, but integrated in the neural parameters as synaptic conductances; and (b) a new transport model based on analytical expressions of the most important transport parameters, which works from sub-pico-level to macro-level, able both to understand existing data and to give new predictions. Complex biological systems are highly dependent on the context, which suggests a “more nature-oriented” computational philosophy.

## 1. Introduction

The great potential of nanobiotechnology is based on the ability to deal with complex hierarchically structured systems from the macroscale to the nanoscale [1]; it requires novel theoretical approaches and the competence of creating models, able to explain the dynamics at such a scale [2,3,4,5]. Geometric and analytical approaches seem to be very promising in all scientific areas, including the study of brain processes.

Deep comprehension and adaptiveness cause a change in the inner brain parameters (conductance of synapses), in order to mimic the outer transformation by the appropriate use of sensors and effectors. The basic mathematical aspects can be illustrated with the use of a toy model related to “Network Resistors with Adaptive Memory” (memristors). Designed by Chua in 1971 [6], only in recent years it has been possible to develop effective realizations [7,8]. The memristor is an electrical circuit with “analogic” properties, able to vary the resistance after a variation in the current and to preserve the last state at the interruption of the energy flow. In a toy model of the brain, this introduces an element of memory that takes into account the enormous non-linear complexity of the homeo-cognitive equilibrium states. This promotes the utility of going back to models of natural computation and therefore of looking at the Turing computation as a “coarse grain” of processes, which are best described by geometric manifolds [9,10,11,12,13].

Technology advancement provides a finer modeling, new solutions, and a capability of active interaction between the environment, machines, and humans and the possibility of not necessarily scaling, as per Moore’s law [14,15,16,17].

Dynamics in the brain are based on transport models; their improvement is a mandatory step in deep comprehension of brain functioning. Advances in analytical modeling can adequately study the nano-dynamics in the brain and lead to interesting ideas for future developments.

Learning experiences produce a “chain action” of signaling among neurons in some areas of the brain, with the modification of neuron connections in particular brain areas, resulting in reorganization. Research on brain plasticity and circuitry also indicates that the brain is always learning, in both formal and informal contexts.

Natural computing refers to observed computational processes and “human-designed/inspired-by-nature” computing. Analyzing complex natural phenomena in terms of computational processes, we reinforce the understanding of both nature and essence of computation. Peculiar to this kind of approach is the use of concepts, principles, and mechanisms underlying natural systems.

This paper aims to highlight some areas of interest for research, combining natural computing, nanotechnology, and brain modelization. It is structured as follows: after an overview of the nanoscience in the brain (Section 2), we consider the technical details of a recently appeared analytical transport model (Section 3). In Section 4, examples of application concerning geometrical images in neural spaces and nano-diffusion together with results are considered, which is followed by conclusions (Section 5).

## 2. Nanoscience in the Brain

Chemical communication and key bio-molecular interactions in the brain occur at the nanoscale; therefore, the idea of taking advantages of nanoscience for advances in the study of brain structure and function is becoming increasingly popular. In the human brain, there are 85 billion neurons and an estimated 100 trillion synapses [18]; as experimental “nano-brain” techniques, we remember:

(1) Snapshots of connections in the brain by making thin physical slices and stacking electron microscopy images (this technique does not provide dynamic and chemical information);
(2) A dynamic voltage map of the brain, dealing with the brain as a close relative of a computer [19,20], with the aim to get to the emergent properties underlying the use and storage of information by a mapping network activity *vs.* single or small numbers of multiple unit recordings;
(3) The attempt to obtain functional chemical maps of the neurotransmitter systems in the brain, for investigating the genetic and chemical brain heterogeneity and the interactions among neurotransmitter systems.

In all these cases, the length scale ranges from the “centimeter” scale (cm) (in mapping brain regions and networks), to the “micrometer” scale (μm) (cells and local connectivity), to the “nanometer” scale (nm) (synapses), to the single-molecule scale [4]. The current ability in performing neurochemical and electrophysiological measurements needs to be miniaturized, sped up, and multiplexed. Electrical measurements at time scales of milliseconds are not complicated, but getting to the nanometer scale and simultaneously making tens of thousands *in vivo* measurements is very difficult. Obtaining dynamic chemical maps at this scale is a bigger challenge, as there are problems in analysis, interpretation, and visualization of data.

## 3. Transport Processes at Nano-Level: Technical Details

Research at the theoretical level help science in all sectors. Recently, a new analytical model that generalizes the Drude-Lorentz and Smith models for transport processes in solid-state physics and soft condensed matter has been used [21,22,23]. It provides analytical time-dependent expressions of the three most important parameters related to transport processes:

(a) The velocities correlation function of a system $<\;\vec{v}\;(t)\;\cdot \;\vec{v}\;(0){>}_{T}$ at the temperature *T*, from which it is possible to obtain the velocity of a carrier at generic time *t*;
(b) The mean squared deviation of position *R*^2^(*t*), defined as $R^{2}(t)=\langle {\left[\vec{R}\;(t)\;-\;\vec{R}\;(0)\right]}^{2}\rangle$, from which the position of a carrier in time is obtainable;
(c) The diffusion coefficient *D*, defined as $D\;(t)=\;(1/2)\;(dR^{2}(t)/dt)$, which gives important information about the temporal propagation of carriers inside a nanostructure [22,23,24].

With this model, it is possible both to fit experimental data, confirming known results, and to discover new features and details. The presence of a gauge factor inside the model allows its use from sub-pico-level to macro-level [25,26].

Starting by the time-dependent perturbation theory, analytical calculation leads to relations for the velocities correlation function, the mean square deviation of position, and the diffusion coefficient of carriers moving in a nanostructure. The general calculation is performed via contour integration in the complex plane. Analytical expressions of the velocities correlation function $<\;\vec{v}\;(t)\;\cdot \;\vec{v}\;(0){>}_{T}$, the mean square deviation of position:

(1) $$R^{2}(t)=\;2\;\int_{0}^{t}dt\prime \text{ ​}\left(t\text{​}-\text{​}t\prime \right)\;\langle \vec{v}\;(t\prime )\;\cdot \;\vec{v}\;(0)\rangle$$

and the diffusion coefficient:

(2) $$D(t)\;=\;\int_{0}^{t}\text{ ​}dt\prime \;\langle \vec{v}\;(t\prime )\;\cdot \;\vec{v}\;(0)\rangle$$

allow a complete dynamical study of carriers.

The classical analytical expressions of the diffusion coefficient *D* are as follows:

(3) $$D\;(t)\;=\;\left(\frac{k_{B}\;T}{m^{*}}\right)\;\left(\frac{\tau }{\alpha_{I}}\right)\;\cdot \left[\exp\;\left(-\;\frac{\left(1\;-\;\alpha_{I}\right)}{2}\;\frac{t}{\tau }\right)\;-\text{ ​}\exp\text{ ​}\left(-\text{ ​}\frac{\left(1\;+\;\alpha_{I}\right)}{2}\;\frac{t}{\tau }\right)\right]$$

(4) $$D\;=\;2\;\left(\frac{K_{B}\;T}{m^{*}}\right)\;\left[\frac{\tau }{\alpha_{R}}\;\sin\;\left(\frac{\alpha_{R}}{2}\;\frac{t}{\tau }\right)\;\exp\;\left(-\;\frac{t}{2\;\tau }\right)\right]$$

The parameters of the model are two real numbers defined in this way:

(5) $$\alpha_{I}^{}\;=\;\sqrt{1\;-4\;\tau^{2}\;\omega_{0}{}^{2}}$$

(6) $$\alpha_{R}^{}\;=\;\sqrt{4\;\tau^{2}\;\omega_{0}{}^{2}\;-\;1}$$

with τ and *ω*_0_ relaxation time and center frequency, respectively, and m^*^ effective mass of the carrier. Generalizations of the model take quantum [23] and relativistic effects [26] into account.

## 4. Examples of Application and Results

**Figure 1:**
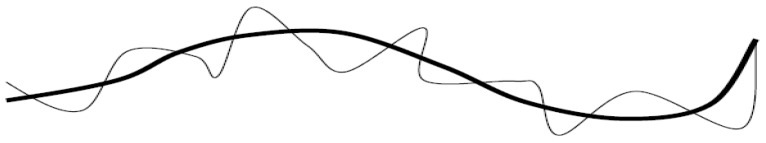
The geodesic as solution of the ODE. With noise, the geodesic is transformed in a more complex structure related to the Fisher information. The total effect is the percolation random geodesic.

(a) An inertial system is used to describe the ordinary differential equation (ODE) types based on standard physical terminology, which is well defined. An example of non-trivial inertial system is the geodesic (kinetic energy) for a mechanical rotatory system with the inertial moment *Ii,j*. In this case, the geodesic is non-inertial, with an invariant given by the respective computable geodesic.
(b) To take into account a minimum of biological plausibility, it is necessary to introduce a simplified membrane model. In a toy membrane just having, for example, three potassium channels (twelve gates), nine open gates can be configured into a variety of topological states, with the possible results that channels are not open, that one is open, or that two are open. Given a vector ***q*** in the channel state, we can compute the probability for the given configuration *q* of states in the channels. Associating a probability to any configuration, there are configurations with very low probability and configurations with high probability. Given the join probability *P*, we compute the variation of the probability with respect to the state *q_j_*. Given the current *i_j_*, we can compute the flux of states $\Phi_{j}$ for the current as a random variable. Assuming the invariant form $\Phi_{j}\text{​}+\lambda \text{​​​ }D_{j}\text{​}P\text{​​ }=\text{​​ }0$, the flux is controlled by the probability in an inverse way, and it is zero when the probability is a constant value. Therefore, we have three different powers: *W*_1_, the ordinary power for the ionic current without noise, *W*_1,2_, the flux of power from current to the noise current, and *W*_*2*_ as power in the noise currents.
(c) About the Fisher information in neurodynamics, computing the average of power as the cost function with a minimum value, we can consider a parametrized family of probability distributions *S* = {*P*(*x*, *t* ,*q*_1_, *q*_2_ ,..., *q_n_*)}, with random variables *x* and *t*, and ***q_i_*** real vector parameters specifying the distribution. The family is regarded as an *n*-dimensional manifold having *q* as a coordinate system; it is a Riemannian manifold, and *G*(*q*) plays the role of a Fisher information matrix. The underlying idea of information geometry is to think the family of probability distributions as a space, each distribution being a “point,” while the parameters *q* plays the role of coordinates. There is a natural unique way for measuring the extent to which neighboring “points” can be distinguished from each other; it has all wished properties for imposing upon a measure of distance, thus keeping “distinguishable” the distance. Considering the well known Kullback-Leibler divergence, with noise equal to zero, the Fisher information assumes the maximum value and the geodesic becomes the classical geodesic. In the case of noise, the information approaches zero, and the cost function is reduced. Resistor networks provide the natural generalization of the lattice models for which percolation thresholds and percolation probabilities can be considered. The geodesic results composed by two parts: the “synchronic and crisp geodesic” and the “noise change of the crisp and synchronic geodesic” (Figure 1).

Therefore, the electric neural activity can be represented in the manifold state space, where the minimum path (geodesic) between two points in the multi-space of currents is a function of the neural parameters as resistors, with or without memory. Simple cases given by electric activity of a little part of the membrane of axons, dendrites, or soma, thus ignoring the presence of the voltage-gated channels in the membrane, can be done. The power is comparable to the Lagrangian in mechanics (Hamilton principle) or the Fermat principle in optics (minimum time). In the context of Freeman’s neurodynamics, we hypothesize that the minimum condition in any neural network gives the meaning of “intentionality”. A neural network changes the reference and the neurodynamics in a trajectory with minimum dissipation of power or geodesic. Therefore, any neural network, or the equivalent electric circuit, generates a deformation of the current space and geodesic trajectories. For every part of the neural network, it is possible to give a similar electric circuit in this way.

(d) In bio-molecules, it is easy to understand that the natural computation is more suitable than the Turing computation. The proposed nanobio approach takes back the attention toward geometric patterns and attractors. As examples of application, we consider a nanomaterial of great interest in nanomedicine, the fullerene in tubular form, *i.e.* carbon nanotubes (CNTs), and we study the behavior of their diffusion using the new proposed analytical model. For the used values, the temperature *T* = 310 K, three values of the parameter $\alpha_{I}^{}$ (0.1, 0.5, 0.9), an average relaxation time $\tau_{av}^{}\;=\;10^{-13}\;s$ (the relaxation time in soft condensed matter takes values of order of 10^−12^–10^−14^ *s*), and two values of m^*^ in relation to the variation of the chiral vector (*n*,*m*) have been fixed [27]:
  (a) (*n*,*m*) = (3,1) → *m*_eff_ = 0.507*m*_e_
  (b) (*n*,*m*) = (7,3) → *m*_eff_ = 0.116*m*_e_
  (*m*_e_ = mass of the electron = 9.109 × 10^−31^ kg)

**Figure 2:**
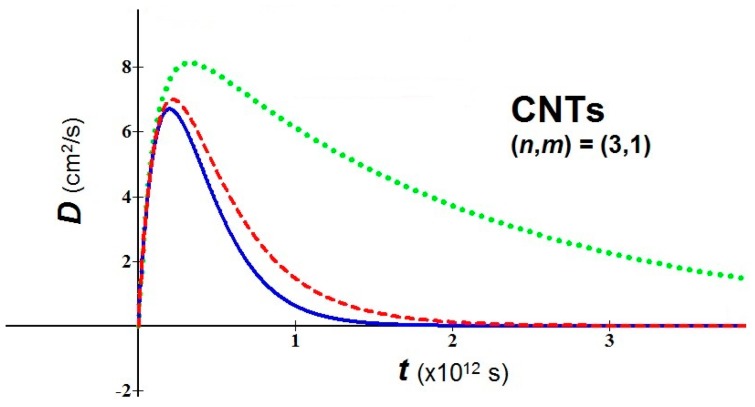
*D vs. t* for CNTs with (*n*,*m*) = (3,1). $\alpha_{I}^{}$ = 0.1 = blue solid line; $\alpha_{I}^{}$ = 0.5 = red dashed line; $\alpha_{I}^{}$ = 0.9 = green dot line.

**Figure 3:**
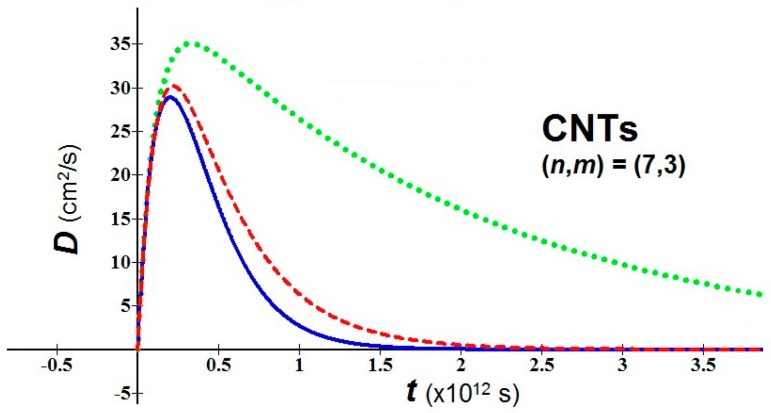
*D vs. t* for CNTs with (*n*,*m*) = (7,3). $\alpha_{I}^{}$ = 0.1 = blue solid line; $\alpha_{I}^{}$ = 0.5 = red dashed line; $\alpha_{I}^{}$ = 0.9 = green dot line.

Figure 2 and Figure 3 show the variation of the diffusion *vs.* time for cases (a) and (b) respectively. It is important to emphasize that the variation of the parameter $\alpha_{I}^{}$ (or $\alpha_{R}^{}$) also implies a variation of the shape of diffusion because of the appearance of $\alpha_{I}^{}$ (or $\alpha_{R}^{}$) in the arguments of exponentials of Equations (3) and (4).

For the peak values of diffusion (in *cm*^2^/*s*) obtainable by Figure 2 and Figure 3, we have:

CTNs (a): 6.74 – 7.05 – 8.15; 

CTNs (b): 28.89 – 30.29 – 35.15

The considered examples clearly show:

(a) The usefulness of a analogic and continuous computation, to which Turing returned with his work on morphogenesis. This kind of computation takes into account the fact that, in these systems, the boundary conditions and the environment are often important; therefore, not everything can be algorithmized;
(b) The usefulness of nano-modeling at this level, which is able to locally provide interesting help in the study of brain dynamics and brain processes.

## 5. Conclusions

In this work we have considered two interesting theoretical approaches related to brain dynamics:

(a) The memory in the neural network, as a non-passive element for storing information: Memory is integrated in the neural parameters as synaptic conductances, which give the geodesic trajectories in the non-orthogonal space of the free states. The optimal non-linear dynamics is a geodesic inside the deformed space that directs the neural computation. This approach provides the ability to mathematically set up the Freeman hypothesis on the intentionality as “optimal emergency” in the “system/environment” relations [28].
(b) A new transport model, based on analytical expressions of the three most important parameters related to transport processes: It holds both for the motion of carriers inside a nanostructure, as considered in this paper, and for the motion of nanoparticles inside the human body, because of an inner gauge factor, allowing its use from sub-pico-level to macro-level. The model can be used to understand and manage existing data, but also to give predictions concerning, for example, the best nanomaterial in a particular situation with peculiar characteristics.

Other possibilities in the direction of a required variation of diffusion concern variations in temperature, variations of the effective mass through doping and chiral vector, the variation of the parameters $\alpha_{I}^{}$ and $\alpha_{R}^{}$ which are functions of the frequency and the relaxation time. The diffusivity of a nano-substance traveling in the human body is an important parameter for a fast diagnosis of possible diseases, potentially leading to a rapid treatment.

We emphasize that the proposed nanobio approach to the brain directs attention to geometric patterns and attractors, which is a general return to analogic-geometric models, made possible by the fine advances in nano-modeling. Despite the simulations using Turing computation, it is clear that the complex biological systems are highly dependent on the context, which suggests a computationally philosophy, more oriented to natural computation [29,30].
